# Evaluation of an Adapted Murine Electrolytic Model of Venous Injury in Wistar Rats: Histopathological Characterization and Challenges in Model Adaptation

**DOI:** 10.7759/cureus.115345

**Published:** 2026-08-28

**Authors:** Emma Eugenia Murariu-Gligor, Bogdan Cordoș, Andreea-Raluca Cozac-Szőke, Alexandru Nicușor Tomuț, Ovidiu S Cotoi, Simona Mureșan

**Affiliations:** 1 Doctoral School of Medicine and Pharmacy, Institution Organizing University Doctoral Studies (IOSUD), George Emil Palade University of Medicine, Pharmacy, Science, and Technology of Târgu Mureș, Târgu Mureș, ROU; 2 Department of Internal Medicine IV, George Emil Palade University of Medicine, Pharmacy, Science, and Technology of Târgu Mureș, Târgu Mureș, ROU; 3 Department of Internal Medicine, Mureș County Clinical Hospital, Târgu Mureș, ROU; 4 Center of Experimental and Imaging Studies, George Emil Palade University of Medicine, Pharmacy, Science, and Technology of Târgu Mureș, Târgu Mureș, ROU; 5 Department of Pathophysiology, George Emil Palade University of Medicine, Pharmacy, Science, and Technology of Târgu Mureș, Târgu Mureș, ROU; 6 Department of Pathology, Mureș County Clinical Hospital, Târgu Mureș, ROU; 7 Faculty of Medicine, George Emil Palade University of Medicine, Pharmacy, Science, and Technology of Târgu Mureș, Târgu Mureș, ROU

**Keywords:** animal model, electrolytic injury, histopathology, venous thrombosis, wistar rats

## Abstract

Introduction

Murine models are widely used in deep vein thrombosis biomedical studies. The principal large-vessel models include ligature-based and free radical thrombosis models (e.g., the ferric chloride or electrolytic models), with the inferior vena cava and femoral vein being the most commonly used vessels. The femoral vein represents an attractive model target due to surgical accessibility and translational relevance, considering its valve-containing structure and thrombus formation in the direction of blood flow after electrolytic injury. Although mice are the most common experimental animals, laboratory rats offer the advantage of larger and more accessible vascular structures. Therefore, this study aimed to adapt an electrolytic injury model in Wistar rats and to perform a preliminary histopathological characterization of the resulting vascular response, including thrombotic findings.

Materials and methods

A prospective, interventional, exploratory study was conducted on 24 female Wistar rats, aged 15 months, following institutional and regulatory approvals. Animals were allocated into sham control (n=4), electrolytic injury control (n=4), and two treated electrolytic injury groups receiving enoxaparin or rivaroxaban (n=8 each). The electrolytic injury consisted of a direct electrical current (3 Volts, 90 seconds) applied to the exposed surface of the femoral vein. Animals in the electrolytic injury control group were euthanized on day 1, while sham and treated animals were euthanized on day 7. Vascular samples underwent histopathological analysis (hematoxylin-eosin and van Gieson elastica stains). Based on recorded findings, two novel semiquantitative scores were developed: the venous wall injury score (VWIS) and the inflammation score (IS). The scores were further compared between treatment groups and between samples with and without detected thrombus.

Results

The main histopathological findings included changes in all vascular layers, occasional necrosis, and mural and/or perivascular inflammatory infiltrates of variable severity. Thrombus presence was detected in one out of four animals from the electrolytic injury control group one day after the procedure, and in five out of 16 animals from the treatment electrolytic injury group seven days after the procedure. Comparison of VWIS and IS at day 7 showed no statistically significant differences between enoxaparin- and rivaroxaban-treated rats. However, VWIS at day 7 was significantly higher in samples containing thrombus compared to those without detected thrombus.

Conclusion

This pilot exploratory study developed and evaluated a modified electrolytic model of venous thrombosis in Wistar rats. The model induced histopathological changes in both the femoral artery and vein, with variable thrombus occurrence, and inflammatory modifications of the venous wall and perivascular tissue. Thrombi detection was associated with a significantly higher VWIS. Further standardization and validation using larger sample groups, different time points, and various model-related conditions (electrical current parameters and therapeutic management), supported by in vivo thrombus visualization techniques, are required for reproducible thrombus induction and reliable treatment comparisons.

## Introduction

Experimental animal models of venous thrombosis have facilitated the understanding of the pathophysiological mechanisms governing thrombus formation and evolution [1], while also supporting therapy development [2].

Murine animal models of disease have been extensively researched in biomedical studies due to their similarities to human anatomy and physiology [3]. Mice and rats represent approximately 95% of laboratory animals [4], as they offer both practical and economic advantages for comparative medicine studies, including low maintenance costs due to their small size, as well as relatively rapid development, short lifespan, and high reproduction rate [3].

There are various rodent experimental models of deep vein thrombosis (DVT) described in the literature, with applicability according to the type of study objectives [5-8]. The main large-vessel models include ligature-based models (e.g., the inferior vena cava (IVC) stasis and stenosis models) and free radical thrombosis models (e.g., the ferric chloride-induced IVC model, the electrolytic IVC model, and the electrolytic femoral vein model) [6,8].

Clinical translation of animal models depends on appropriate model selection according to the intended clinical scenario, by clearly mentioning the flow state of the vessel, the type of injury employed, and the method of model evaluation [9]. Given the multitude of studies reporting different variations of the main proposed models, together with the lack of standardized protocols, an expert consensus was published in 2019, meant to guide the selection and application of mouse animal models of venous thrombosis [8]. The recommendations for selecting the most appropriate model considered the specific objectives envisioned, as each model presents both advantages and disadvantages [7,8]. For instance, the IVC ligation model consists of completely interrupting the blood flow, therefore limiting studies aiming to research the effect of therapeutic agents on thrombus resolution [7]. Conversely, models that preserve vessel patency (such as stenosis or electrolytic injury models) enable the evaluation of therapeutic interventions [7] (e.g., thrombolytic or anticoagulant therapies). Additionally, the electrolytic model facilitates thrombus formation in the direction of blood flow and can also be applied to the femoral vein; this enhances the clinical translation of the animal model, as the femoral vein contains valves, therefore better mimicking the pathophysiological mechanisms observed in human venous thrombosis [8].

The 2019 expert consensus synthesized the literature available at that time and offered valuable guidance, including supplementary material containing detailed descriptions of the recommended surgical techniques for each animal model [8]. A specific mouse protocol for inducing venous thrombosis through femoral vein electrolytic injury was provided by using the blunt end of a microsurgical needle (70-75-micron diameter) connected to a direct current source to deliver the electrical injury to the exposed part of the femoral vein [8]. However, the implementation of this model raises technical challenges, including the requirement for precise microsurgical manipulation and the anatomical constraints related to the small size of the animals and their corresponding vascular structures. Furthermore, the recommendations were not tailored to other rodent species. Alternatively, the use of laboratory rats has potential advantages (e.g., larger body size and more easily accessible vascular anatomy, which may facilitate procedural handling) [3]. Although mice are the most commonly used animal in biomedical studies [4], laboratory rats are widely used in cardiovascular research [3] because they offer several practical advantages for in vivo studies evaluating therapeutic interventions, including the ability to obtain larger blood volumes, perform repeated blood sampling at multiple time points, and facilitate tissue harvesting. However, the exact requirements necessary to adapt the previously described mouse protocol to Wistar rat anatomy and physiology to achieve reproducible thrombus formation require further evaluation.

Our study aimed to investigate the adaptation of the electrolytic murine model of venous thrombosis to Wistar rats. The primary objective was to characterize the resulting vascular changes, including thrombotic findings. A secondary exploratory objective was to assess the histopathological findings following two different anticoagulant treatments. Additional analyses of blood parameters derived from the same experimental population are reported separately and are outside the scope of the current manuscript.

## Materials and methods

Study design

A prospective, interventional, exploratory study was implemented in 2023, following ethics committee and institutional approval (Decision No. 1881, dated 12 October 2022, issued by the Scientific Research Ethics Committee of George Emil Palade University of Medicine, Pharmacy, Science, and Technology of Târgu Mureș, and Project Authorization No. 58, dated 6 March 2023, issued by the Animal Health and Welfare Official Control Service, Veterinary and Food Safety Authority Mureș, Romania). The design of the study was in accordance with the Animal Research Reporting of In Vivo Experiments (ARRIVE) criteria.

A total of 24 female Wistar rats (*Rattus norvegicus*), aged 15 months, obtained from the university's animal facility, were assigned to the study. Inclusion criteria consisted of being clinically healthy, with normal results of baseline complete blood count (CBC) analysis. Exclusion criteria, consisting of clinical evidence of illness and/or abnormal hematological findings on CBC testing, were not met.

Animals were housed in groups, according to their experimental group assignment. Animal identification was conducted by ear notching. Food and water were provided ad libitum, with a 12/12h light/dark cycle. Animals were handled by trained personnel, and invasive procedures were conducted under gaseous anesthesia and analgesia with respect to humane endpoints, to reduce unnecessary suffering. Predefined humane endpoints included signs of severe pain or distress, impaired mobility, marked weight loss, and severe post-procedural complications. No animals met the humane endpoint criteria throughout the study.

Randomization was not applicable, as the 24 animals were sequentially allocated into experimental groups. The study design included a sham control group (n=4) for baseline vascular analysis, an electrolytic injury model control group (n=4) used to assess the feasibility of the procedure, and an electrolytic injury group receiving anticoagulant treatment (n=16). The treated group was further subdivided into two anticoagulation arms: a fractionated heparin (enoxaparin) and a direct oral anticoagulant (rivaroxaban) (n=8 each).

In the treated electrolytic injury group, anticoagulation was started one day after the procedure and continued for six days, with once-daily administration according to group allocation. Enoxaparin (Clexane) was administered subcutaneously at a body weight-adjusted dose (10.5 mg/kg/day). Rivaroxaban (Xarelto) was administered orally using Nutella-based pellets prepared in-house according to a standardized formulation containing starch and lactose as excipients, with identical-size pellets (281 mg of formulation, containing 0.75 mg of rivaroxaban) administered daily, after prior habituation of animals to plain Nutella to ensure voluntary consumption of the treatment formulation.

Following the intervention, animals were euthanized at the prespecified endpoint for their respective experimental group: day 1 for the electrolytic injury model control group, and day 7 for the sham control and treated electrolytic injury groups. Euthanasia was performed by terminal cardiac puncture under deep anesthesia, followed by tissue collection for histopathological assessment. Femoral vein specimens were collected from all animals. Additionally, femoral artery samples were collected from the sham control and electrolytic injury model control groups to allow histopathological comparison of adjacent vascular structures. Blinding was not applicable to procedure-related steps; however, it was applied to histopathological examination.

The primary outcomes were the histopathological features of the vascular samples following electrolytic injury, including venous wall injury, inflammatory changes, and the detection of thrombus at the time of euthanasia. Secondary exploratory outcomes were histopathological findings according to anticoagulant regimen and thrombus status at endpoint.

The experimental animal model

The electrolytic model implemented in this study was adapted from the recommendations outlined in the 2019 consensus on mouse models of venous thrombosis [8], as no standardized rat protocol comparable to the mouse model was available in the literature at the time of study design. The surgical procedure involved applying the blunt end of a needle - connected to a direct current source - to the exposed surface of the femoral vein. The previously described procedure stated that in mice this process leads to electrolytic deposition of iron from the needle onto the surface of the vein, inducing the formation of a non-occlusive thrombus, progressing in the downstream direction from the site of electrical application [8]. To accommodate the anatomical and physiological characteristics of the selected animal model (Wistar rats), the technique was modified from the original mouse protocol by increasing the applied voltage (from 1.5 V to 3 V) and extending the duration of current delivery to 90 seconds, while remaining within the recommended range of 1 to 180 seconds. The detailed procedure is described in the next paragraphs.

The surgical procedure was performed under inhalation anesthesia with isoflurane (Anesteran). Induction was achieved using 5% isoflurane in combination with oxygen at a flow rate of 0.4-0.6 L/min, administered in an induction chamber. Following induction, anesthesia was maintained with 2-3% isoflurane delivered via a nasal cone, accompanied by oxygen at a flow rate of 0.2 L/min.

Following fur removal from the inguinal region and the medial aspect of the left hind limb, the animal was placed in dorsal recumbency. The surgical site was disinfected using povidone-iodine (Betadine), and a sterile surgical drape was applied to isolate the left hind limb. A skin incision was then performed, followed by blunt dissection to expose the femoral neurovascular bundle. The femoral vein and artery were identified, individually dissected, and gently separated using fine suture threads.

After preparation of the materials required for the electrolytic model - including a 10/0 microsurgical needle (3.8 mm length, 70 μm diameter) (MN103, SMI AG, Belgium), two surgical forceps, and a 3-V continuous current power source - a closed electrical circuit was established. The procedure was performed under microscopic guidance. The negative terminal of the power source was first connected to a forceps clamping the overlying skin to close the electrical circuit. Subsequently, the blunt end of the microsurgical needle, secured in a second pair of forceps connected to the positive terminal, was gently applied to the exposed surface of the femoral vein, and a continuous 3-V potential was applied for 90 seconds; the actual current (amperage) delivered was not measured.

Upon completion of the procedure, the skin incision was closed by approximating the wound edges and applying a medical-grade tissue adhesive - Surgibond (SMI AG), followed by the application of a veterinary aluminum spray to provide additional wound protection. Postoperative analgesia was ensured by the subcutaneous administration of a standard dose of 0.1 mL tramadol (50 mg/mL).

A similar protocol was applied to the sham control group, consisting of skin incision, dissection, and isolation of the femoral neurovascular bundle, followed by surgical wound closure without application of the electrolytic injury.

Histopathological analysis

Tissue specimens were fixed in 10% neutral buffered formalin for 24 hours at room temperature. Following fixation, the samples were processed through standard tissue processing and subsequently embedded in paraffin blocks. The collected samples, approximately 5 mm in length, were oriented for transverse sectioning to allow evaluation of the entire venous lumen. Serial sections of 3 µm thickness were cut from the paraffin blocks using a semi-automated microtome and mounted on glass slides.

For each sample, three serial histological sections were obtained and examined at approximately 100 µm intervals to reduce the possibility of missing small, focal thrombi due to the plane of sectioning. Routine hematoxylin and eosin (H&E) staining was performed using an automated staining system. Elastic van Gieson staining was additionally applied on selected sections to evaluate vascular morphology and the integrity of elastic fibers in the venous and arteriolar walls.

Histopathological evaluation focused on vascular structures, including assessment of vessel wall integrity, endothelial alterations, inflammatory infiltrates, perivascular changes, mural necrosis, and intraluminal thrombosis.

All stained sections were examined independently by two board-certified pathologists (A.R.C.S. and O.S.C.) who were blinded to the experimental group, and any discrepancies were resolved by joint review and consensus. Histopathological assessment was performed using a Zeiss Axio Lab A1 light microscope (Carl Zeiss Microscopy GmbH, Jena, Germany). Digital photomicrographs were captured at multiple magnifications (×5, ×10, ×20, and ×40) for comprehensive analysis and documentation.

Following the descriptive histopathological characterization, two original, study-specific semiquantitative scoring systems were developed by the authors to standardize the assessment of venous injury and inflammatory changes across specimens: the venous wall injury score (VWIS) and the inflammation score (IS). To the best of our knowledge, no similar scoring systems have been previously described or published.

The VWIS was established by assigning points for every modification observed involving the main vessel wall layers (intima, media, and adventitia), resulting in a maximum score of 4 points/sample (Table 1).

**Table 1:** Semiquantitative venous wall injury score (VWIS) calculation derived from the study’s descriptive histopathological characterization

VWIS	Criteria	Points
Endothelial injury	Partially denuded endothelium	1 p
	Reactive changes in endothelial cells	1 p
Media and adventitial injury	Edema of the smooth muscle layer	1 p
	Mural necrosis	1 p

Similarly, the IS was calculated by assigning points according to the type, location, and severity of the inflammatory infiltrate, resulting in a maximum score of 8 points (Table 2).

**Table 2:** Semiquantitative inflammation score (IS) calculation derived from the study’s descriptive histopathological characterization

IS	Criteria	Points
Inflammatory infiltrate type	Active, with neutrophils	1 p
	Chronic, lymphoplasmacytic	1 p
	Presence of multinucleated giant cells	1 p
Inflammatory infiltrate location	Perivascular	1 p
	Within the vessel wall	1 p
Inflammatory infiltrate severity	Reduced	1 p
	Moderated	2 p
	Abundant	3 p

Statistical analysis

Data were organized using Microsoft Office 365 Excel (Microsoft Corp., Redmond, WA, USA). Statistical analyses were performed using MedCalc® Statistical Software version 23.5.0 (2026; MedCalc Software Ltd, Ostend, Belgium). Semiquantitative histopathological scores were expressed as median with interquartile range (IQR). Histopathological scores were compared between independent groups using the Mann-Whitney U test. Fisher’s exact test was used to compare thrombus frequency between the two anticoagulant treatment groups. Statistical significance was set at p-values less than 0.05.

## Results

In vivo observations

**Figure 1:**
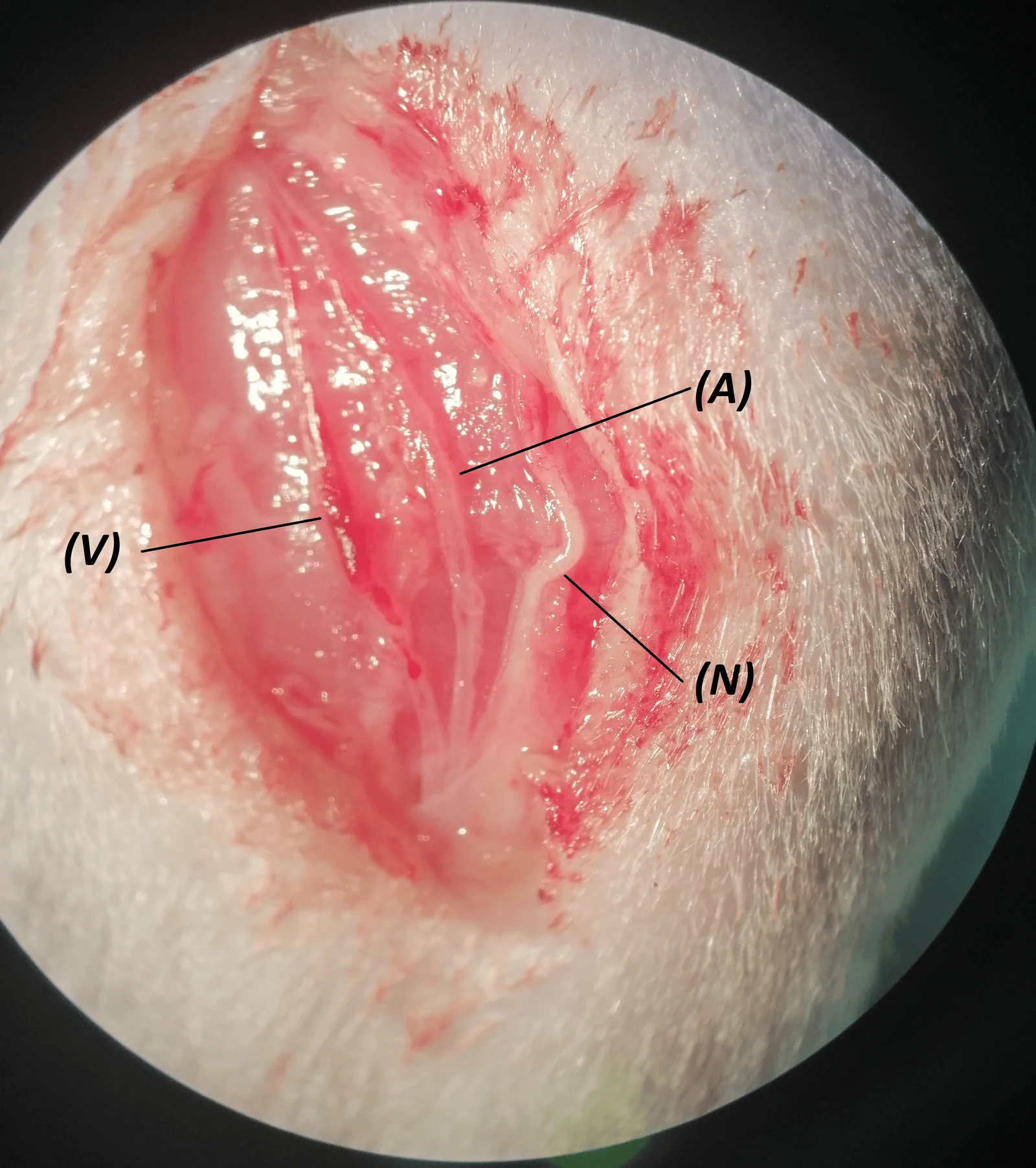
Intraoperative microscopic aspect of the exposed surface of the left femoral vein following the application of electrical current (V) Femoral vein, with thrombus visible inside the vessel; (A) femoral artery; (N) femoral nerve.

After the application of electrical current to the exposed venous wall, microscopic examination demonstrated partial evidence of the expected iron deposition from the needle onto the venous surface, with subsequent thrombus development (Figure 1).

Histopathological modifications in the sham control group

**Figure 2:**
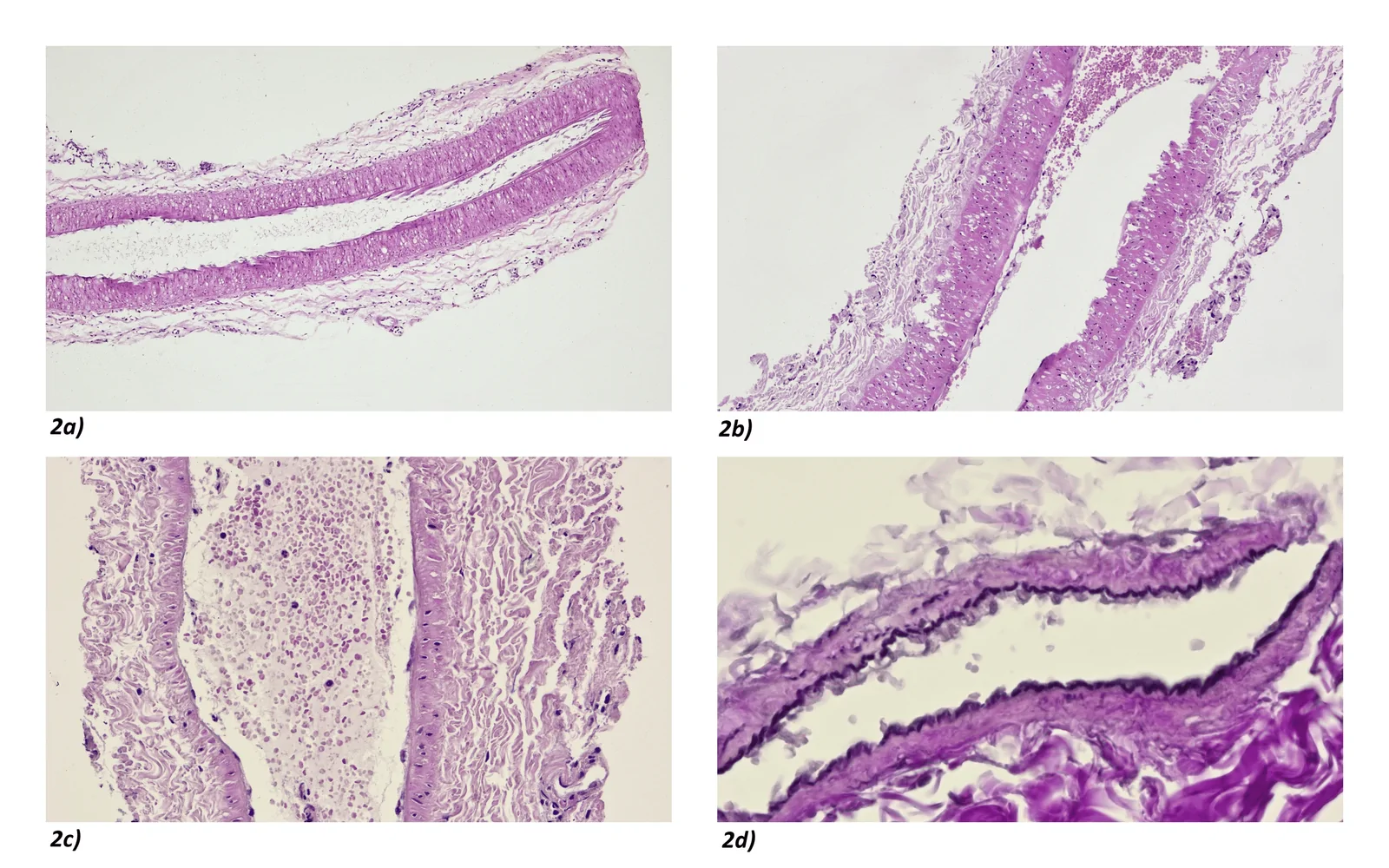
Histopathological findings in the arteriolar specimens of sham group, showing normal arteriolar wall structure The vessel wall layers are intact, with unremarkable perivascular tissue; the van Gieson elastica stain confirms the continuity of elastic fibers in vascular walls, without any fragmentation. (a) Hematoxylin and eosin (H&E) stain, 5x magnification; (b) H&E stain, 10x magnification; (c) H&E stain, 20x magnification; (d) Van Gieson elastica stain, 20x magnification.

**Figure 3:**
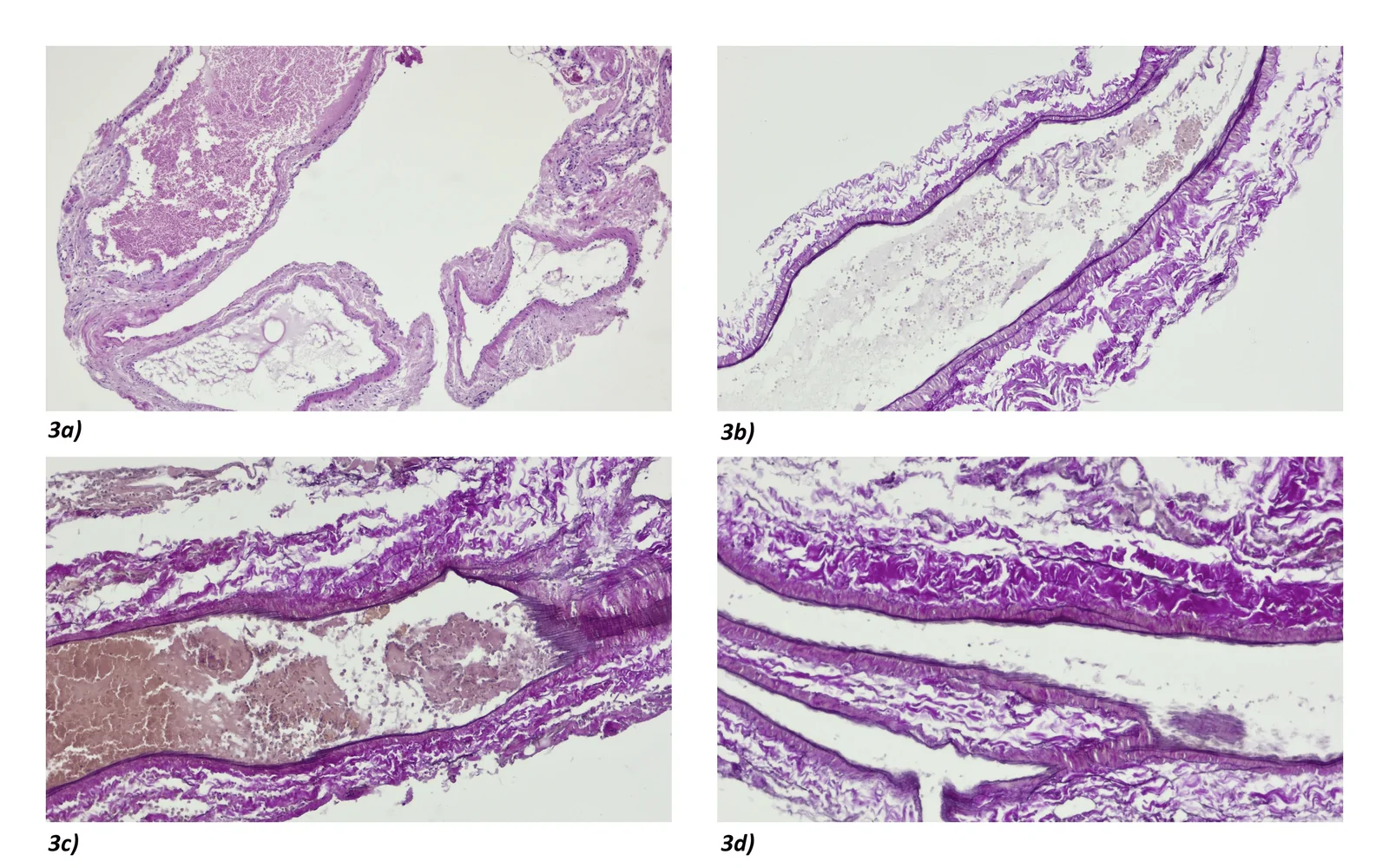
Histopathological findings in the venous specimens of sham group, showing normal venous wall structure The vessel wall layers are intact, with unremarkable perivascular tissue. In van Gieson elastica stain, the elastic fibers are colored dark brown/black, confirming the continuity of elastic fibers in the vascular wall, without any fragmentation. (a) Hematoxylin and eosin (H&E) stain, 10x magnification of normal venous wall structure, with venous stasis. (b) Van Gieson elastica stain, 5x magnification of normal venous wall. (c) Van Gieson elastica stain, 10x magnification of normal venous wall and venous stasis. (d) Van Gieson elastica stain, 20x magnification of normal venous wall.

In sham control group samples, both arteriolar (Figure 2) and venous (Figure 3) samples displayed preserved wall architecture, with intact wall layers and no evidence of mural necrosis, inflammatory infiltrate, or structural damage. The perivascular connective tissue was unremarkable, without inflammatory cells or pathological alterations. The endothelial lining was continuous and intact in both vessel types, without signs of denudation or reactive endothelial changes. Elastic van Gieson staining demonstrated well-preserved, continuous elastic fibers within venous and arteriolar walls, showing a regular and organized arrangement, without fragmentation or loss of elastic integrity (Figures 2-3).

Histopathological modifications in the electrolytic injury group

**Figure 4:**
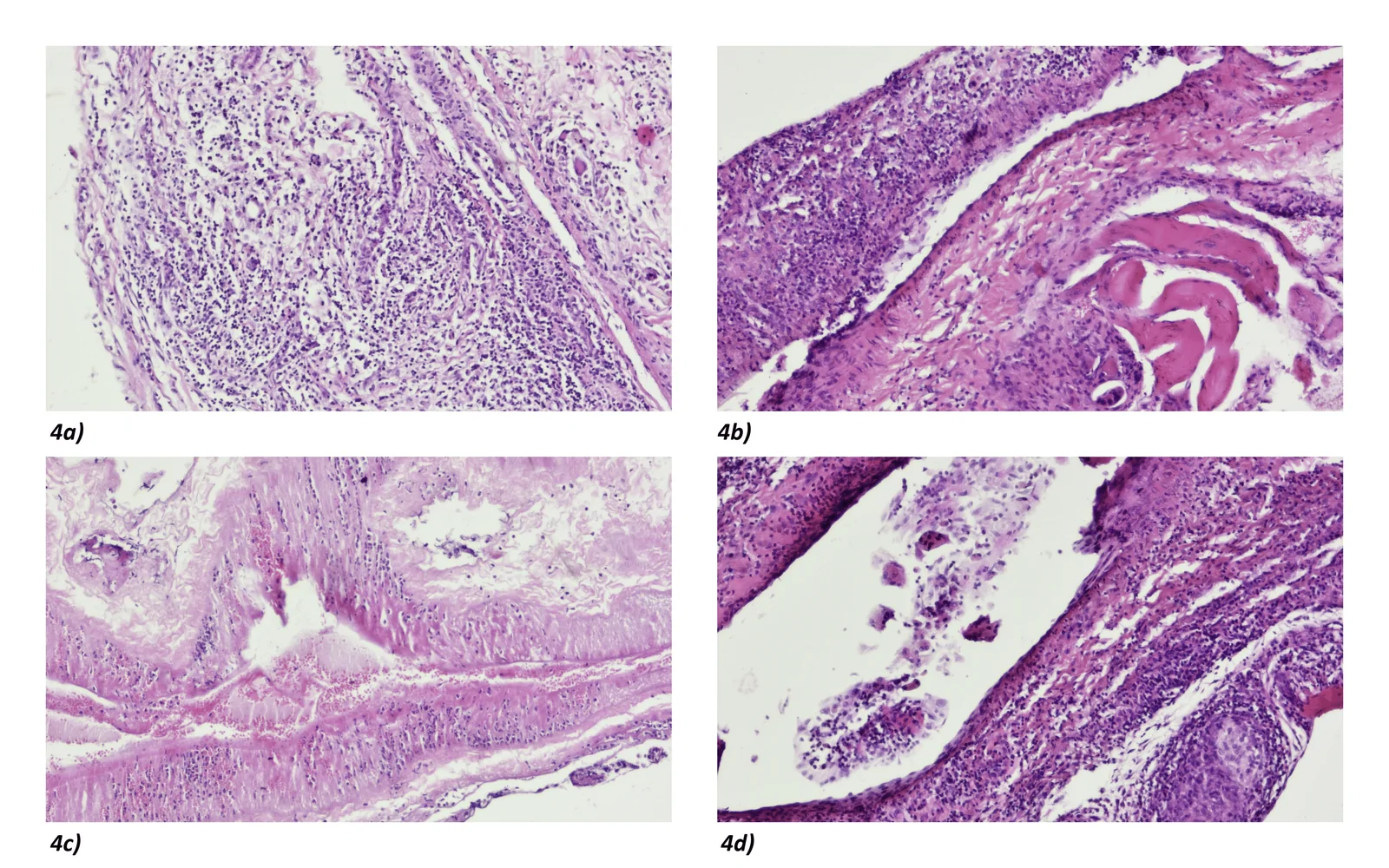
Histopathological findings in the venous specimens of electrolytic injury group in hematoxylin and eosin (H&E) stain (a) H&E stain, 20x magnification of the venous wall, displaying a mixed inflammatory infiltrate in the perivascular connective tissue. (b) H&E stain, 20x magnification of the venous wall, displaying vessel wall necrosis and a mixed inflammatory infiltrate in the perivascular connective tissue. (c) H&E stain, 20x magnification of the venous wall, displaying vessel wall necrosis and abundant granulocytic inflammatory infiltrate in the venous wall. (d) H&E stain, 20x magnification of the venous wall, displaying intraluminal thrombus and a mixed inflammatory infiltrate in the perivascular connective tissue.

On H&E staining, several venous walls exhibited severe structural injury, characterized by extensive mural necrosis and a dense mixed inflammatory infiltrate involving the full thickness of the vessel wall and extending into the perivascular connective tissue. The inflammatory infiltrate was predominantly composed of numerous neutrophilic granulocytes, with admixed inflammatory cells (Figure 4).

**Figure 5:**
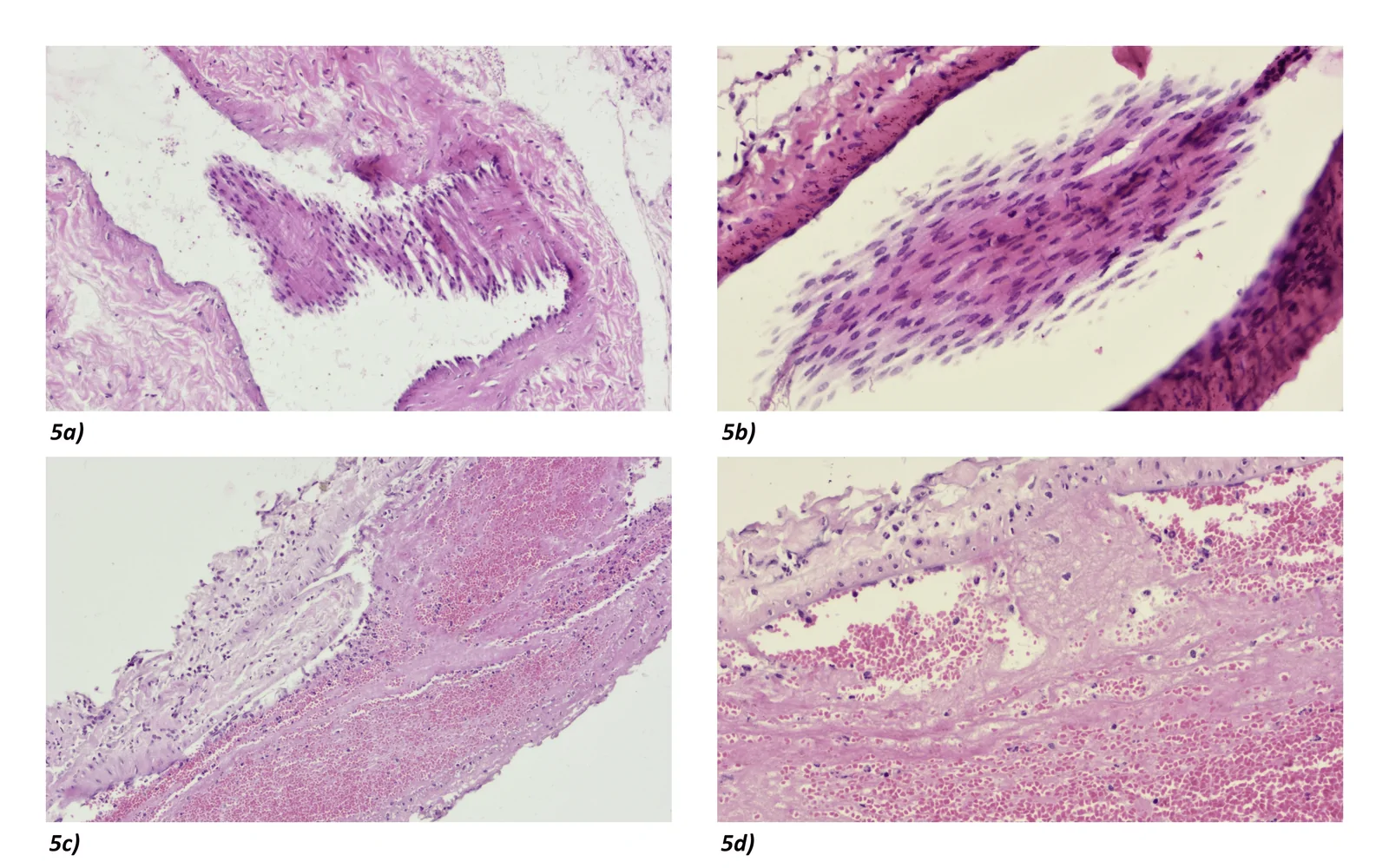
Histopathological findings in the venous specimens of electrolytic injury group in H&E stain, displaying presence of thrombus in several venous specimens (a) H&E stain, 10x magnification of the venous wall, displaying intraluminal thrombus adherent to the wall. (b) H&E stain, 20x magnification of the venous wall and lumen, displaying intraluminal thrombus. (c) H&E stain, 10x magnification of the venous wall and lumen, displaying venous stasis and thrombi containing fibrin, leukocytes, and erythrocytes. (d) H&E stain, 20x magnification of the venous wall and lumen, displaying venous stasis and thrombi containing fibrin, leukocytes, and erythrocytes, respectively, with an associated mixed inflammatory infiltrate in the perivascular tissue.

In several venous segments (6/20), thrombotic material was detected. Mural thrombi were firmly adherent to the damaged vascular wall and were composed of fibrin-rich material with entrapped erythrocytes and inflammatory cells, consistent with venous-type thrombosis (Figure 5).

The endothelial lining was partially denuded in affected areas, while in regions where it was preserved, endothelial cells displayed reactive changes, including cellular swelling and nuclear enlargement, reflecting endothelial injury and activation.

**Figure 6:**
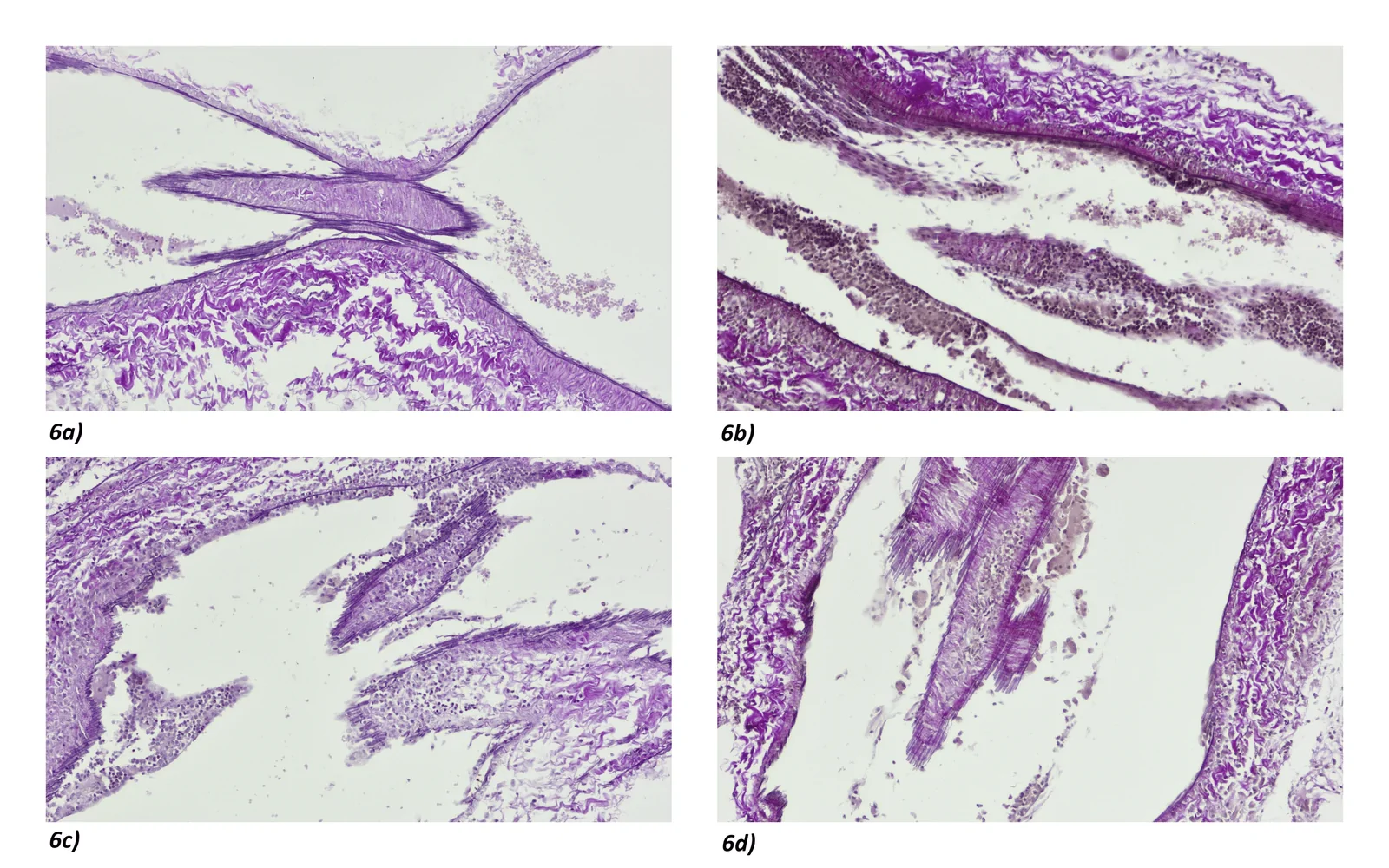
Histopathological findings in the venous specimens of electrolytic injury group in van Gieson stain (a-b) 10x magnification of the venous wall and lumen, displaying intraluminal thrombus. (c-d) 10x magnification of the venous wall and lumen, displaying intraluminal thrombus adherent to the vessel wall.

Elastic van Gieson staining was performed to better evaluate the integrity of the vascular wall and elastic fibers. Elastic van Gieson staining highlighted severe disruption and fragmentation of elastic fibers within the venous wall, with focal loss of the normal elastic architecture, corresponding to areas of mural necrosis and inflammatory destruction. In less affected areas, residual elastic fibers were preserved, although displaying an irregular and disrupted arrangement (Figure 6).

Detailed histopathological description of vascular samples in the electrolytic injury control group is provided in Table 3.

**Table 3:** Histopathological changes observed on hematoxylin and eosin (H&E) staining in the vascular wall and intraluminal thrombus detection in the electrolytic injury control group Animal ID refers to the identifier of each animal, e.g., C1 - animal number 1 from the electrolytic injury control group.

Animal ID (electrolytic injury control group)	Vessel type (artery/vein)	Modifications of the vascular wall structure		Inflammatory infiltrate characterization				Thrombus (assessed at day 1)
		Endothelial changes	Changes in media + adventitia	Type	Multinucleated giant cells	Location	Quantity	
C1	Artery	Partially denuded endothelium + reactive changes in endothelial cells	Edema of the smooth muscle layer, necrosis	Active, with neutrophils	Absent	In the vessel wall + perivascular	Moderate	Not detected
C1	Vein	Partially denuded endothelium + reactive changes in endothelial cells	Edema of the smooth muscle layer	Active, with neutrophils	Absent	Perivascular	Reduced	Detected
C2	Artery	Partially denuded endothelium + reactive changes in endothelial cells	Edema of the smooth muscle layer, necrosis	Active, with neutrophils	Absent	Perivascular	Reduced	Not detected
C2	Vein	Partially denuded endothelium + reactive changes in endothelial cells	Absent	Active, with neutrophils	Absent	In the vessel wall + perivascular	Abundant	Not detected
C3	Artery	Partially denuded endothelium + reactive changes in endothelial cells	Edema of the smooth muscle layer, necrosis	Active, with neutrophils	Absent	Perivascular	Reduced	Not detected
C3	Vein	Partially denuded endothelium + reactive changes in endothelial cells	Absent	Active, with neutrophils	Absent	In the vessel wall + perivascular	Abundant	Not detected
C4	Artery	Partially denuded endothelium + reactive changes in endothelial cells	Edema of the smooth muscle layer, necrosis	Active, with neutrophils	Absent	Perivascular	Abundant	Not detected
C4	Vein	Partially denuded endothelium + reactive changes in endothelial cells	Absent	Active, with neutrophils	Absent	In the vessel wall + perivascular	Moderate	Not detected

Detailed histopathological descriptions of venous samples from the electrolytic injury treated groups are provided in Table 4 (for enoxaparin-treated arm) and Table 5 (for rivaroxaban-treated arm).

**Table 4:** Histopathological changes observed on hematoxylin and eosin (H&E) staining in the vascular wall and intraluminal thrombus detection in the enoxaparin-treated group Animal ID refers to the identifier of each animal, e.g., E1 - animal number 1 from the enoxaparin-treated group.

Animal ID (enoxaparin-treated)	Modifications of the venous wall structure		Inflammatory infiltrate characterization				Thrombus (assessed at day 7)
	Endothelial changes	Changes in media + adventitia	Type	Multinucleated giant cells	Location	Quantity	
E1	Partially denuded endothelium + reactive changes in endothelial cells	Absent	Active, with neutrophils	Present	Perivascular	Reduced	Not detected
E2	Partially denuded endothelium	Absent	Chronic, lymphoplasmacytic	Absent	Perivascular	Reduced	Not detected
E3	Partially denuded endothelium + reactive changes in endothelial cells	Absent	Active, with neutrophils	Present	In the vessel wall + perivascular	Abundant	Not detected
E4	Partially denuded endothelium + reactive changes in endothelial cells	Edema of the smooth muscle layer	Active, with neutrophils	Absent	In the vessel wall + perivascular	Abundant	Detected
E5	Partially denuded endothelium + reactive changes in endothelial cells	Edema of the smooth muscle layer	Active, with neutrophils	Present	In the vessel wall + perivascular	Abundant	Detected
E6	Absent	Absent	Chronic, lymphoplasmacytic	Present	Perivascular	Reduced	Not detected
E7	Partially denuded endothelium + reactive changes in endothelial cells	Edema of the smooth muscle layer	Active, with neutrophils	Present	In the vessel wall + perivascular	Abundant	Detected
E8	Partially denuded endothelium	Absent	Chronic, lymphoplasmacytic	Absent	Perivascular	Reduced	Not detected

**Table 5:** Histopathological changes observed on hematoxylin and eosin (H&E) staining in the vascular wall and intraluminal thrombus detection in the rivaroxaban-treated group Animal ID refers to the identifier of each animal, e.g., R1 - animal number 1 from the rivaroxaban-treated group.

Sample (rivaroxaban-treated)	Modifications of the venous wall structure		Inflammatory infiltrate characterization				Thrombus (assessed at day 7)
	Endothelial changes	Changes in media + adventitia	Type	Multinucleated giant cells	Location	Quantity	
R1	Partially denuded endothelium + reactive changes in endothelial cells	Edema of the smooth muscle layer, necrosis	Chronic, lymphoplasmacytic	Absent	Perivascular	Reduced	Not detected
R2	Partially denuded endothelium + reactive changes in endothelial cells	Absent	Chronic, lymphoplasmacytic	Absent	Perivascular	Reduced	Not detected
R3	Partially denuded endothelium + reactive changes in endothelial cells	Absent	Chronic, lymphoplasmacytic	Absent	Perivascular	Moderate	Not detected
R4	Partially denuded endothelium + reactive changes in endothelial cells	Absent	Active, with neutrophils	Present	In the vessel wall + perivascular	Abundant	Not detected
R5	Partially denuded endothelium + reactive changes in endothelial cells	Edema of the smooth muscle layer, necrosis	Active, with neutrophils	Present	Perivascular	Moderate	Not detected
R6	Partially denuded endothelium + reactive changes in endothelial cells	Edema of the smooth muscle layer	Active, with neutrophils	Absent	Perivascular	Moderate	Detected
R7	Partially denuded endothelium + reactive changes in endothelial cells	Absent	Active, with neutrophils	Present	In the vessel wall + perivascular	Abundant	Not detected
R8	Partially denuded endothelium + reactive changes in endothelial cells	Edema of the smooth muscle layer	Active, with neutrophils	Absent	Perivascular	Moderate	Detected

Use of semiquantitative scoring systems

When comparing the electrolytic injury control group on day 1 to the electrolytic injury treatment group on day 7, no significant differences in median VWIS or IS were observed (Table 6).

**Table 6:** Comparison of venous wall injury score (VWIS) and inflammation score (IS) between the electrolytic injury control group on day 1 and the electrolytic injury treatment group on day 7 post-intervention IQR: interquartile range

Outcome	Electrolytic injury control group, day 1 post-intervention (n=4)	Electrolytic injury treatment group (enoxaparin + rivaroxaban), day 7 post-intervention (n=16)	Mann-Whitney U test for independent samples		
			U	Z	p
VWIS, median (IQR)	2 (2-2.5)	2 (2-3)	29.5	-0.252	0.801
IS, median (IQR)	5.5 (4-6)	4 (3.5-7)	31.5	-0.048	0.961

Furthermore, when comparing individual treatment arms from the electrolytic injury treatment group, no significant differences in median VWIS or IS were observed between the enoxaparin- and rivaroxaban-treated arms on day 7 post-intervention (Table 7).

**Table 7:** Comparison of venous wall injury score (VWIS) and inflammation score (IS) between the electrolytic injury groups treated with enoxaparin or rivaroxaban, assessed on day 7 post-intervention IQR: interquartile range

Outcome	Electrolytic injury treatment group - enoxaparin (n=8)	Electrolytic injury treatment group - rivaroxaban (n=8)	Mann-Whitney U test for independent samples		
			U	Z	p
VWIS, median (IQR)	2 (1-3)	2.5 (2-3.5)	19.0	-1.426	0.154
IS, median (IQR)	5 (3.5-7)	4 (3.5-6)	28.0	-0.436	0.663

However, when comparing groups with and without thrombus detected at day 7 post-intervention, median VWIS was significantly higher in samples with histopathological evidence of thrombus than in those without detected thrombus (Mann-Whitney U test, p = 0.038). No significant difference in median IS was observed between the two groups (Table 8).

**Table 8:** Comparison of venous wall injury score (VWIS) and inflammation score (IS) according to histopathological evidence of thrombus at day 7 post-intervention IQR: interquartile range

Outcome	Electrolytic injury treatment group - thrombus detected (n=5)	Electrolytic injury treatment group - thrombus not detected (n=11)	Mann-Whitney U test for independent samples		
			U	Z	p
VWIS, median (IQR)	3 (3-3)	2 (1.25-2)	10.0	-2.071	0.038
IS, median (IQR)	6 (4-7)	4 (3-6.5)	17.0	-1.236	0.217

Thrombus detection at day 7 did not differ significantly between the enoxaparin and rivaroxaban electrolytic injury-treated groups (Fisher’s exact test, p = 1.000) (Table 9).

**Table 9:** Comparison of histologically detected thrombus between the electrolytic injury groups treated with either enoxaparin or rivaroxaban at day 7 post-intervention

Electrolytic injury treatment arm	Thrombus detected, n (%)	Thrombus not detected, n (%)	Fisher’s exact test, p-value
Enoxaparin-treated	3 (37.5)	5 (62.5)	1.000
Rivaroxaban-treated	2 (25.0)	6 (75.0)	

## Discussion

When choosing a murine animal model of venous thrombosis that can maintain venous blood flow while generating a thrombus that can be followed up dynamically, two main options were considered for this study: the IVC stenosis model and the femoral vein electrolytic model. The IVC stenosis model of thrombosis has the advantage of targeting a larger-caliber vein, but requires a midline laparotomy, followed by manipulation of intra-abdominal contents, which is associated with higher intra-operative stress, inflammation, and longer postoperative recovery. By comparison, the electrolytic model, although involving a smaller vein, benefits from easier surgical access via a groin incision, resulting in a less invasive procedure, reduced operative stress, and improved postoperative recovery. The electrolytic model was therefore preferred for balancing technical feasibility with animal welfare considerations. However, as this was the first implementation of the electrolytic model at our center, the learning curve of the microsurgical model adaptation may have contributed to procedural variability.

Previous studies on murine models with successful thrombus induction provided conflicting results regarding sex-related differences in thrombus size. A previous study on an IVC ligation model in Sprague-Dawley rats (*R. norvegicus*) showed no statistically significant difference between male and female rats in thrombus mass, harvested at one day and three days post-ligation [10]. However, another study on an electrolytic IVC model of thrombosis in mice showed significantly larger thrombi in males versus females at two, six, and 14 days post-model induction, with differences attributed to sex-related particularities in vascular anatomy and response to inflammation [11]. Given the small sample size and to avoid potential bias due to sex-related differences in the response to the electrolytic stimulus, the current study included only female rats.

Our study protocol was based on the research published by Cooley et al. on the mouse electrolytic femoral vein model of thrombosis, which he refined in several papers [12-15]. The model was first described in 2005, when it was applied to transgenic mice to generate non-occlusive thrombus, visualized by scanning electron microscopy, with peak growth at 30 minutes but regression by 60 minutes [12]. The authors then refined the model and applied it to the mouse carotid artery and femoral vein by adapting the voltage and time of current application to each type of vessel. The electrolytic injury based on iron deposition led to clot formation, with the thrombus peaking in growth at more than 10 minutes, and further gradual dissolution by microemboli. By using in vivo fluorescence and fluorophore-labeled platelets and fibrin, he was able to describe the mechanisms of thrombus formation and characterize its structure - platelets accumulating as a large mass attached to the injury site, with fibrin localized predominantly at the border, appearing to serve as a stabilizing structure to prevent thrombus microembolization [13].

However, in our study, thrombus detection was inconsistent, and the feasibility of the proposed adapted protocol to successfully induce thrombosis in Wistar rats was limited. After applying the electrolytic injury protocol, thrombi were histopathologically confirmed in only one of four model control animals, one day after the intervention, and in five out of 16 treated animals, seven days after the intervention. With this limitation in mind, identified thrombi were histopathologically similar to the above descriptions. The histopathological findings included mural thrombi firmly attached to the lesion site (Figure 5a), intraluminal thrombi (Figure 4d and Figure 5b), or intraluminal venous stasis and thrombi containing fibrin, leukocytes, and erythrocytes (Figures 6c-6d). Regardless of thrombus confirmation or not, perivascular inflammatory infiltrates were visible (Figures 4a, 4d), consisting of predominantly polymorphonuclear neutrophilic granulocytes. These findings are also supported by previous models of venous thrombosis in mice where both the venous wall and the thrombus were infiltrated with neutrophils in the acute phase (from thrombus induction up to day 2 or 3), with a shift towards neutrophils and monocytes in the subacute phase (around day 6) [6]. In our model control tissue samples, harvested one day after the procedure, the inflammatory infiltrate was exclusively polymorphonuclear granulocytic, rich in neutrophils, while at day 7 in the treatment group samples, the character of the inflammatory infiltrate varied: either polymorphonuclear granulocytic (mostly neutrophils) or lymphoplasmacytic, with occasional multinucleated giant cells. The presence of multinucleated giant cells has also been previously described in an endovascular porcine model of ilio-caval venous thrombosis, when they were observed inside the mature thrombus at three, four, and five weeks post-procedure [16].

The standardized femoral vein electrolytic model of thrombosis in mice offers several advantages, including the presence of venous valves and thrombus propagation in the direction of blood flow. However, one of its principal limitations is the small thrombus size. In 12-16-week-old male C57BL/6 mice, histomorphometric thrombus volume at day 1 has been reported to range from 0.015 to 0.140 mm^3^, depending on the applied current duration and voltage [8]. In our rat model, the H&E staining identified thrombus presence in only six out of 20 venous specimens (one of four electrolytic injury controls, respectively five out of 16 electrolytic injury-treated). Given the lack of a standardized protocol for rodent species other than mice and the absence of in vivo imaging techniques to confirm thrombus formation, it cannot be determined whether failure to identify thrombi on histopathological examination reflected lack of thrombus formation, spontaneous embolization, treatment-related thrombus resolution, or technical limitations related to tissue sampling and histological sectioning of such small thrombi.

Thrombus persistence may be influenced by both the experimental protocol and the characteristics of the animal model, as suggested by different findings reported by Cooley et al. In adult C57BL/6 mice, application of a 3-V electrolytic injury to the femoral vein for 90 seconds resulted in persistent thrombi at both one and seven days after the procedure, followed by fibrotic remodeling with luminal stenosis at 14 and 28 days [15]. In contrast, a previous study using a modified model that combined electrolytic injury with reduced venous flow in adult female CD-1 mice reported thrombus resolution after four days in the preserved-flow model, while venous wall remodeling and fibrotic transformation of the thrombus were persistent out to 28 days in the reduced venous flow model variation [14]. Our adapted electrolytic injury protocol similarly used a 3-V, 90-second application; however, the actual current delivered to the exposed venous vessel was not quantified. Interestingly, in the electrolytic injury control group, in which arterial tissue samples were also evaluated, histopathological evidence of injury was observed in both venous and arterial walls, although the electrical stimulus was applied only to the venous exposed surface. This finding highlights the need for further technical adaptation of the model to improve vascular specificity, including optimization of the surgical needle size, optimization of electrical parameters (voltage and amperage) to limit arterial damage while maintaining a sufficient degree of venous injury for a more consistent thrombus occurrence, and assessment of thrombus evolution at additional time points. Within this context, comparison of untreated and anticoagulated animals at serial time points may provide further insight into the factors contributing to thrombus persistence.

Cooley also observed that heparin administered in high therapeutic dose led to a diminished fibrin composition (approximately 10x less) in both venous and arterial thrombi [13]. Given the limited number of histologically confirmed thrombi, meaningful comparisons between the enoxaparin and rivaroxaban treatment groups regarding thrombus fibrin content could not be performed.

The proposed semi-quantitative histopathological scores, namely the VWIS and the IS, are original to this study. According to our data, median VWIS and IS did not statistically differ between the electrolytic injury control group (untreated) and the combined (enoxaparin and rivaroxaban) treated group. However, the groups were assessed at different temporal endpoints: the electrolytic injury control group was assessed on day 1 post-intervention (to allow for preliminary procedural assessment), while the treatment group was assessed on day 7 post-intervention, which may lead to confounding time- and procedure-related effects. On day 7, no statistically significant differences in VWIS or IS were observed between enoxaparin- and rivaroxaban-treated rats. However, these findings are exploratory and should be interpreted cautiously, as the small sample size, lack of randomization, and absence of a comparable untreated day 7 control group limit definitive conclusions regarding therapeutic comparisons. Within the combined enoxaparin- and rivaroxaban-treated group at day 7, VWIS was significantly higher in samples containing thrombus than in those without detected thrombus. Given the small sample sizes, however, this finding should be interpreted as an association rather than evidence of causation. Furthermore, as the present study is a pilot preliminary investigation, the proposed scores require further standardization and validation before their broader application.

While ex vivo assessment of venous thrombosis models relies on thrombus weight and histological analysis, their terminal nature does not allow for longitudinal analysis of thrombus evolution [17]. Several imaging modalities have been described for in vivo longitudinal assessment of murine venous thrombosis models: intravital microscopy [18,19], high frequency ultrasonography [18] (including Doppler imaging, contrast-enhanced ultrasonography, or ultrasound elastography [18]); high performance imaging techniques such as magnetic resonance imaging (MRI) venography, computed tomography (CT) application (e.g. contrast-enhanced micro-CT), fusion imaging modalities (e.g., CT fluorescence molecular tomography), and radionucleotide imaging - for instance positron emission tomography CT (PET-CT) or single-photon emission CT (SPECT) [18]. A major limitation of the current study was the unavailability of these imaging techniques for monitoring possible thrombus formation and evolution over time. Consequently, it could not be determined whether the low rate of histologically confirmed thrombi at both day 1 and day 7 reflected failure of thrombus formation, thrombus resolution under anticoagulant treatment, embolization, or technical limitations of histopathological detection.

Broadly speaking, successful implementation of an animal model requires careful consideration of important study design and methodological aspects. These include whether the selected model is appropriate for the proposed hypothesis, the relevant anatomical, physiological, and pathophysiological characteristics of the chosen species, the technical expertise and refinement required to establish the procedure, and the available methods for in vivo or ex vivo assessment of the experimental outcome.

Study limitations

Given that this was the first attempt, to the best of our knowledge, to adapt the electrolytic injury protocol to Wistar rats, the presented study aimed to provide preliminary histopathological characterization of the vascular response, including thrombus detection, rather than to validate a reproducible thrombosis model or determine treatment efficacy. The main limitations of our study include both study design and methodological limitations.

Study-design limitations include the small sample size, the use of older female rats, sequential rather than randomized allocation, and the lack of additional exploratory control groups (e.g., an untreated electrolytic injury group, evaluated at the same endpoint as the treatment groups, or groups exposed to different electrical parameters). Additionally, the different prespecified euthanasia endpoints may introduce time-related confounders. These limitations are relevant because the non-randomized allocation and small group sizes limit the statistical power and the strength of between-group comparisons of the two anticoagulant regimens, and the absence of a day 7 untreated electrolytic injury group further limits interpretation of treatment-associated effects. As a result, comparisons between anticoagulant groups should be considered exploratory rather than confirmatory.

Methodological limitations also affect the interpretation and reproducibility of these findings. The technical challenges and learning curve associated with the initial implementation and adaptation of this animal model at our facility, together with the arterial injury observed in model control samples, indicate that further protocol refinement and validation are needed. Further evaluation should consider not only the applied voltage but also the actual current delivered at the vessel surface, tissue electrical resistance, and electrode-tissue contact, as well as potential operator-related variability. These factors were not fully standardized or quantified in the present study and may have contributed to variability in the vascular response to electrolytic injury.

Thrombus assessment was also subject to methodological limitations. Thrombus detection relied on histopathological examination at the prespecified study endpoint, without in vivo imaging to monitor thrombus formation and evolution, or quantitative assessment of thrombus volume. Vascular specimens were embedded in paraffin, with the vein oriented to facilitate transverse sectioning and evaluation of the vascular lumen; three serial histological sections were examined in H&E staining for each sample, aiming to reduce the likelihood of missing small focal thrombi because of the plane of sectioning. Nevertheless, the histopathological sampling strategy was not fully standardized, and small or focal thrombi may therefore have remained undetected. All sections were independently assessed by two board-certified pathologists, with discrepancies resolved by consensus; however, formal interobserver agreement was not quantified. The VWIS and IS scores used in this study are original, developed specifically for the present histopathological assessment, and therefore have not been independently validated. Their reproducibility and discriminative value require further evaluation under standardized experimental conditions.

Taken together, these design and methodological limitations make it difficult to draw definitive conclusions about thrombus occurrence, treatment effects, or the reproducibility of the proposed experimental model. The present findings should therefore be considered a preliminary characterization of the vascular response to an adapted electrolytic injury protocol. The inconsistent detection of thrombus limits its interpretation as a reproducible thrombosis model.

## Conclusions

The present pilot, exploratory study provides an initial adaptation of a previously described mouse electrolytic model of venous thrombosis to Wistar rats. The adapted protocol induced histopathological alterations in both the femoral arteries and veins, involving the endothelial, medial, and adventitial layers, together with inflammatory infiltration of the venous wall and surrounding perivascular tissue. Thrombi were detected in a subset of animals, indicating a variable thrombotic response under the applied experimental conditions. Inflammatory infiltrates were observed in all samples undergoing the electrolytic procedure, but with different degrees of severity, as quantified by the proposed histopathological scoring systems. Exploratory comparison of the two anticoagulation regimens did not identify significant differences in VWIS or IS between groups. Thrombus detection was associated with a higher VWIS, suggesting that the extent of venous wall involvement may reflect differences in thrombus-associated tissue response. However, these findings represent preliminary characterization of the vascular response, rather than validation of a reproducible thrombosis model or evidence of treatment efficacy.

Further protocol refinement and validation are required before the model can be considered suitable for reproducible thrombus formation or reliable treatment comparisons. Future studies should incorporate larger sample sizes, additional control groups, multiple assessment timepoints, systematic evaluation of electrical parameters, and standardized quantitative and qualitative assessment of thrombus formation, ideally complemented by in vivo imaging.
